# Autoimmune Disorders and Thrombophilia in Pathologic Pregnancies: Management Throughout the Full Gestation

**DOI:** 10.3390/biomedicines14051143

**Published:** 2026-05-18

**Authors:** Rui Gao, Lang Qin

**Affiliations:** 1 Reproductive Medical Center, Department of Obstetrics and Gynecology, West China Second University Hospital, Sichuan University, Chengdu 610041, China; ruigao@scu.edu.cn; 2 Key Laboratory of Birth Defects and Related Diseases of Women and Children, Ministry of Education, West China Second University Hospital, Sichuan University, Chengdu 610041, China; 3 Development and Related Diseases of Women and Children Key Laboratory of Sichuan Province, West China Second University Hospital, Sichuan University, Chengdu 610041, China

**Keywords:** autoimmune disorders, thrombophilia, antiphospholipid syndrome, recurrent pregnancy loss, pathologic pregnancy

## Abstract

Pathologic pregnancies including recurrent pregnancy loss, stillbirth, early-onset pre-eclampsia and early-onset fetal growth restriction form a continuous spectrum throughout gestation and have attracted wide attention. Autoimmune disorders and associated acquired thrombophilia are key etiological factors. However, because of the complicated associations between various clinical manifestations, laboratory examinations and treatments, the management of pathologic pregnancies with autoimmune disorders and associated acquired thrombophilia are difficult. This viewpoint article presents a comprehensive full gestation management strategy emphasizing early identification and multidisciplinary management to improve pregnancy outcomes in these patients. Future research should focus on novel biomarkers, therapeutic methods and crosstalk mechanisms between autoimmune disorders and thrombophilia to optimize clinical strategies.

## 1. Introduction

With the decline of fertility in recent years, pathologic pregnancies such as recurrent pregnancy loss (RPL), stillbirth and early-onset early placental insufficiency manifesting as early-onset preeclampsia, fetal growth restriction (FGR) and oligohydramnios (<34 weeks of gestation) have received widespread attention. RPL is defined as the loss of two or more pregnancies, affecting 1–2% of women of child-bearing age [1]. Genetic factors, anatomical factors, endocrinal disorders, autoimmune disorders and thrombophilia are all associated with RPL [2]. Stillbirth is a severe pathologic pregnancy induced by various systemic diseases, pregnancy complications and fetal abnormalities. Early-onset pre-eclampsia is part of a range of pregnancy complications that stem from issues with placental development. This spectrum, known as early-onset placental insufficiency, also encompasses stillbirth, early-onset FGR, oligohydramnios and other manifestations of fetal hypoxemia [3]. It worth mentioning that the above pathologic pregnancies are not isolated incidents but part of a continuum throughout the full gestation. Some etiologies and pathogeneses are shared in these different manifestations of pathologic pregnancies. For instance, during the pathogenesis of early-onset pre-eclampsia, trophoblast dysfunction caused by various genetic and immunological factors was presented in the first and second trimesters of gestation and induced sallow placentation. Then, reduced placental perfusion was observed and subsequently lead to RPL, stillbirth, FGR, and even pre-eclampsia, determined by the degree of hypoperfusion [4].

Autoimmune disorders and thrombophilia are important causes of RPL, stillbirth and early-onset placental insufficiency (pre-eclampsia, FGR and other manifestations of fetal hypoxemia). The management of pathologic pregnancies with autoimmune disorders and associated acquired thrombophilia is difficult, due to complicated clinical manifestations, laboratory examinations and therapeutic methods. Recognizing and managing these patients with autoimmune disorders or associated acquired thrombophilia early may reduce the risk of pregnancy complications and improve final pregnancy outcomes. Here, we try to construct a comprehensive management strategy throughout the full gestation for autoimmune disorders and associated acquired thrombophilia in patients with pathologic pregnancies, to help improve outcomes in the present or following pregnancy.

## 2. Autoimmune Disorders in Pathologic Pregnancies

Autoimmune disorders are a group of conditions mediated by various autoantibodies, including antinuclear antibody (ANA), antiphospholipid antibody (aPLs), anti-SSA/Ro and anti-SSB/La antibodies and the spectrum of ANA. Usually, the treatment of autoimmune disorders should be based on a definitive diagnosis. However, some early stages of autoimmune disorders may trigger pathologic pregnancies for pregnant women and are always ignored in clinical practice. For these conditions, the clinical management strategy is controversial. To help in clinical management, we summarize autoimmune disorders into three stages: positive autoantibodies, undifferentiated connective tissue disease (UCTD) and definitive autoimmune diseases.

### 2.1. Positive Autoantibodies

ANAs are a diverse group of autoantibodies targeting macromolecular components in the cell nucleus and binding to DNA, RNA and proteins, as well as complexes of nucleic acids with proteins [5]. According to previous published meta-analysis based on retrospective cohort and case–control studies, positive ANA has been identified to increase the risk of RPL, stillbirth and various pregnancy complications, but the ANA titer cut-off values were not consistent in different studies [6,7]. Roughly, the positive rate of ANA in RPL patients is as high as 20.0%, more than 8.3% in control women [7]. However, low ANA titers of 1:80 or 1:160 were non-specific and do not warrant pharmacological treatment in clinical practice. In our opinion, the detection and intervention of ANA in pathologic pregnancies were controversial at present, but for those who excluded more prevalent etiologies (especially genetic causes, anatomical factors and endocrine disorders), ANA detection by indirect immunofluorescence on Hep-2 cells and solid-phase assays should also be considered to recognize the potential cause. At present, the interventional strategy for ANA positivity in pathologic pregnancies lacks evidence support; immunomodulatory treatment based on hydroxychloroquine (HCQ) and low-dose corticosteroids may be experimental used, but a detailed strategy still needs to be explored in future clinical trials.

A special condition is that, within the spectrum of ANA, the presence of positive anti-SSA/Ro and anti-SSB/La antibodies has been demonstrated to increase the risk of fetal congenital heart block (CHB) and neonatal lupus [8]. As reported, the estimate positive rates of anti-SSA/Ro antibodies were 5% for spontaneous abortion, 4% for perinatal death, 3% for FGR and 7% for CHB [9]. So, for pregnancies with positive anti-SSA/Ro and anti-SSB/La antibodies (cut-off values are different in different detective platforms) management is recommended [10]. For pregnancies with positive anti-SSA/Ro or anti-SSB/La antibodies, immunomodulatory treatment based on HCQ and low-dose corticosteroids should be employed during the full gestation. Fetal echocardiography should also be performed biweekly from 16 to 28 weeks of gestation to detect fetal CHB. If CHB is detected, corticosteroids, even IVIG, should be initiated promptly [10].

### 2.2. Undifferentiated Connective Tissue Disease

UCTD is considered a pre-state of various autoimmune diseases. Recently, it has gained attention as an independent disease, especially given the limited progression from UCTD to definite autoimmune diseases. The prevalence of RPL, FGR and gestational hypertension was higher in UCTD pregnant women than in healthy pregnancies [11]. As reported in a multicenter cohort, the miscarriage, FGR and pre-eclampsia rates in UCTD patients are 20.1%, 2.7% and 2.2%, respectively [11]. Although pathologic pregnancies were not traditionally recognized as manifestations of UCTD, RPL associated with positive ANA (excluding other causes) has been referred to as “pregnancy loss-associated UCTD” [12], and should be managed throughout the full gestation. The therapeutic strategies of UCTD in pregnant women are controversial. HCQ, a low dose of aspirin and low-dose corticosteroids were all tried in previous studies. However, a previous observational study found that prednisone plus aspirin did not show improved pregnancy outcomes in RPL patients with UCTD [13]. Immunomodulatory treatment based on HCQ with low-dose corticosteroids was used for pathologic pregnancies with UCTD in a three-arm, multicenter, open-label randomized controlled trial, but the final results were not published [14].

### 2.3. Autoimmune Diseases

Systemic lupus erythematosus (SLE) and Sjögren’s syndrome (SS) are classic ANA-associated autoimmune diseases. SLE is a complex syndrome with variable clinical features [15], significantly increasing the risks of RPL, stillbirth, pre-eclampsia, fetal CHB and neonatal lupus [16]. Disease activity, irreversible damage and presence of aPLs are the high-risk factors for adverse pregnancies in SLE patients [16]. SS is a systemic autoimmune disease that presents with a wide spectrum of clinical manifestations and autoantibodies. Pregnancies complicated with SS carry a significantly higher risk for fetal CHB and neonatal lupus, primarily due to the presence of anti-SSA/Ro and anti-SSB/La antibodies [17].

For pregnancy combined with these autoimmune diseases, a multidisciplinary team (MDT) comprising specialists in reproductive medicine, obstetrics and rheumatology may provide insights into management. The specialist in rheumatology should estimate the patients to guide the conditions of pregnancy. Pregnancy may be considered if the SLE has been stable for at least six months, with low-dose oral corticosteroids (equivalent to ≤15 mg/d of prednisone), 24 h urinary protein quantification < 0.5 g, and no significant organ damage, and if gonadotoxic and teratogenic drugs have been discontinued for the required duration [18]. Throughout the full gestation, both the pregnancy complications and the disease activity need to be monitored. Immunomodulatory treatment based on HCQ, low-dose corticosteroids, even intravenous immunoglobulin (IVIG), represents the basis for treating these patients. If the disease re-flares, tacrolimus, cyclosporine A, high-dose corticosteroids, IVIG and even plasma exchange can be considered [18]. Fetal development should also be monitored throughout the entire pregnancy; Doppler ultrasound examinations of the umbilical artery, uterine artery, ductus venosus and fetal middle cerebral artery represent a common strategy to estimate fetal perfusion.

We must be clear enough about the purpose of screening and managing these conditions. For patients presenting with severe pre-eclampsia, placental insufficiency, or stillbirth, these interventions may not reverse the outcome of the present pregnancy. The main purpose of screening is to guide subsequent pregnancies. For patients with a history of RPL, identifying and intervening in these issues during the full gestation can help improve pregnancy outcomes.

## 3. Thrombophilia in Adverse Pregnancies

Thrombophilia refers to a group of conditions that increase the risk of abnormal blood clotting, leading to venous and arterial thromboembolism [19]. Since pregnancy is already a prothrombotic state, pregnant women with thrombophilia face an even higher risk of maternal–fetal interface micro-thrombosis and subsequent pathologic pregnancies. The body’s hemostatic balance is composed of four components: endothelial cells and the coagulation system, anticoagulation system and fibrinolytic system. Any reason leading to excessive activation of endothelial cells and the coagulation system, or excessive inhibition of the anticoagulation and fibrinolytic systems, is regarded as thrombophilia.

Thrombophilia can be either inherited or acquired. Inherited thrombophilia stems from genetic mutations in genes involved in or regulating clotting factors. The common inherited thrombophilia includes factor V Leiden, prothrombin G20210A, antithrombin deficiency, protein C deficiency, and protein S deficiency. Diagnostic evaluation usually involves functional assays for protein activities or DNA analysis. Inherited thrombophilia was shown to increase the risk of RPL and other pathologic pregnancies [20,21]. At present, only RPL patients were recommend for inherited thrombophilia screening in international guidelines [2]. In pathologic pregnancies, acquired thrombophilia attracted more widespread attention. Autoimmune conditions, especially those involving aPLs and antiphospholipid syndrome (APS), are the most typical acquired thrombophilias.

### 3.1. Autoimmune Disorders and Acquired Thrombophilia: The Close Association Mediated by Antiphospholipid Antibodies

aPLs serve as the key link between autoimmune disorders and acquired thrombophilia. aPLs, including lupus anticoagulant (LA), anticardiolipin antibody (aCL) and anti-ß2 glycoprotein I antibody (aß2GPI), are the most important autoantibodies for increasing the risk of various pathologic pregnancies [22]. β2-glycoprotein I (β2GPI), primarily synthesized by hepatocytes, is a major target for aPLs and is expressed on the surface of decidual endothelial cells and trophoblasts during pregnancy, making them susceptible to aPL binding.

The mechanisms by which aPLs induce pathologic pregnancies involve thrombophilia, inflammatory damage and maternal–fetal immune dysregulation [23]. Endothelial cell injury and dysfunction are the initial steps in aPL-induced coagulation system activation and thrombosis, collagen exposure, von Willebrand factor (vWF), tissue factor (TF) and adhesion molecules take part in this pathway [23]. Platelets and monocytes are also the targets of aPLs in the pathogenesis of thrombosis. The recruitment of neutrophils also promotes local immunothrombosis by the formation of neutrophil extracellular traps [24]. These complex mechanisms lead to local microthrombus formation, disrupting maternal–fetal blood supply and causing adverse pregnancies.

Emerging evidence highlights that aPLs can trigger inflammatory damage at the maternal–fetal interface by binding to endothelial cells and trophoblasts, releasing various inflammatory factors and chemokines through multiple pathways [25]. aPLs also suppress trophoblast proliferation, invasion, and human chorionic gonadotropin expression, disrupting placental formation and spiral artery remodeling and reducing placental perfusion [26]. Maternal–fetal immune tolerance is crucial for maintaining pregnancy, with decidual immune cells (natural killer cells, macrophages, T cells, B cells) recognizing and protecting embryo-derived trophoblasts from immune attack and promoting spiral artery remodeling and trophoblast invasion [27]. Abnormal immune activation and infiltration in the decidua are characteristic of many pathologic pregnancies related to placental maldevelopment, especially RPL, early-onset pre-eclampsia and early-onset FGR [28]. Our previous studies identified significant macrophage infiltration and endothelial cell activation in decidua of patients with aPL-induced miscarriage, with increased inflammatory factors and chemokines [29]. Chemokine receptor expression on endothelial cells accelerates immune cell infiltration and subsequent inflammation in the decidua [30].

### 3.2. Antiphospholipid Antibodies and Antiphospholipid Syndrome

The pathologic pregnancies widely recognized to be associated with aPLs include RPL, stillbirth, early-onset pre-eclampsia and early-onset FGR. The prevalence of aPLs in RPL patients were different between different studies, with a meta-analysis of RPL populations demonstrated the broad ranges (1.8–36.5% LAC, 2–88.1% aCL) [31]. A multicenter retrospective case–control study reported that 9.6% of stillbirths after 20 weeks of gestation tested positive for aCL or aβ2GPI, a prevalence 3–5 times higher than in healthy pregnancies [32]. A study also found that LA-positive pregnancies had a 4.6-fold increased risk of FGR compared to controls [33]. All patients with the above pathologic pregnancies should be screened for LA, aCL and aß2GPI as early as possible to assess the risk of subsequent pregnancies. Antiphospholipid syndrome (APS) is a thrombo-inflammatory disease propelled by circulating aPLs that cause vascular thrombosis and obstetrical complications; the classification of APS must be established based on the presence of aPLs and clinical manifestations [22,34]. Patients with positive aPLs and those diagnosed with APS represent different stages of autoimmune disorders. However, given the pathogenicity of aPLs, all these patients should receive standardized management throughout the full gestation.

Given the complex clinical manifestations and the dual mechanisms involving immunity and coagulation, management of patients with positive aPLs or APS should follow a comprehensive approach throughout the full gestation addressing both anticoagulation and immune modulation. Low-dose aspirin (LDA) and/or prophylactic-dose low-molecular-weight heparin (LMWH) is the standard treatment recommend by various international guidelines for these patients but is shown to have insufficient effects in clinical practice [35]. According to the European League Against Rheumatism recommendations for management of APS, increasing LMWH dose to a therapeutic dose is the most common practice if the prophylactic dose of LMWH fails [35]. Immunomodulatory treatment based on HCQ and low-dose corticosteroids, even IVIG, has also been tried in refractory APS patients, with satisfactory effects [36,37,38]. Although the indications for combining anticoagulation therapy with immunomodulatory treatment are not yet clear, for high-risk patients, such as refractory APS, the early addition of immune modulation therapy may be beneficial.

### 3.3. Acquired Thrombophilia Management in Other Autoimmune Disorders

In addition to APS, in pregnancy with other autoimmune disorders, there should also be a focus on the risk of acquired thrombophilia. Endothelial cell injury may be the underlying mechanism for acquired thrombophilia caused by autoimmune disorders [39,40]. Activated partial thromboplastin time (APTT) is useful for recognizing the acquired thrombophilia in autoimmune disorders. If APTT is prolonged, a mixed test by phospholipid should be performed to identify the causes of prolonged APTT, and the Silica clotting test and diluted Russell Viper Venom time (dRVVT) specific test were also necessary for recognizing the presence of LA [41]. Recently, some novel coagulation molecular markers, such as thrombomodulin, thrombin–antithrombin complex, plasmin–antiplasmin complex and tissue-type plasminogen activator–plasminogen activator inhibitor-1 complex, can more sensitively reflect endothelial cell damage and the activation of the coagulation and fibrinolytic systems. However, these novel markers are not widely used in clinical practice because the detection methods lack standardization and unified reference intervals in pregnant women and are unavailable in many laboratories. Anticoagulant therapy of LDA with or without LMWH should be considered throughout the full gestation in those with autoimmune disorders and acquired thrombophilia according to individual risks, to prevent subsequent pathologic pregnancies and thrombosis [35].

## 4. Conclusions

In conclusion, we summarized a management strategy for autoimmune disorders and associated acquired thrombophilia in patients with pathologic pregnancies, emphasizing comprehensive care throughout the full gestation. Early identification and management of autoimmune disorders are crucial to prevent subsequent pathologic pregnancies. Patients with positive aPLs and APS, representing the intersection of autoimmune disorders and acquired thrombophilia, require dual management with anticoagulation and immune modulation therapies. For other autoimmune disorders, screening and treatment for acquired thrombophilia are also vital. Future research should focus on elucidating novel biomarkers, therapeutic methods and mechanisms of crosstalk between autoimmune disorders and acquired thrombophilia in pathologic pregnancies, offering deeper insights for comprehensive management strategies.

## Data Availability

No new data were created or analyzed in this study. Data sharing is not applicable to this article.
